# Reverse Bernheim Phenomenon – A True Enigma

**DOI:** 10.1016/j.rmcr.2025.102250

**Published:** 2025-07-01

**Authors:** Evan Wasserman, Luis Wulff, Debabrata Bandyopadhyay, Ricardo Restrepo-Jaramillo, Raheel Qureshi, Jose Herazo-Maya, Monirul Islam

**Affiliations:** University of South Florida, Geisinger Health, United States

**Keywords:** Pulmonary arterial hypertension, Reverse bernheim syndrome, Interventricular septum, RV dysfunction, PAH specific treatment, LVOT obstruction, Bernheim syndrome, Ventricular interdependence, Diastolic dysfunction

## Abstract

A 70-year-old man was admitted with progressive dyspnea and exercise-induced syncopal episodes. His past medical history was significant for heart failure with preserved ejection fraction (HFpEF) and pulmonary arterial hypertension (PAH). He was treated with ambrisentan, sildenafil and selexipag. Due to progressive symptoms, he underwent right heart catheterization (RHC). Compared to his prior, this showed improvement in mean pulmonary arterial pressure (mPAP) but worsened pulmonary capillary wedge pressure (PCWP). He was therefore titrated off selexipag and continued on other medications. Despite this his symptoms continued to worsen.

Resting echocardiography demonstrated normal left ventricular (LV) systolic function with right ventricle (RV) dilatation and dysfunction; no shunt across the interatrial septum was detected. During a stress echocardiogram he had an episode of syncope with associated hypotension and oxygen desaturation. The images confirmed dramatic RV dilatation at peak exercise along with leftward bulging of the septum leading to compression of the left ventricular outflow tract (LVOT) - “the reverse Bernheim phenomenon”. The RV size and function recovered rapidly at rest. The patient's PAH therapy was subsequently escalated with addition of intravenous treprostinil, which led to improvement of exercise capacity and resolution of syncopal episodes. A repeat stress echocardiogram revealed resolution of the acute exercise-induced RV dilatation and septal bulging without compressing the LVOT.

## 1 Background

**Fig. 1:**
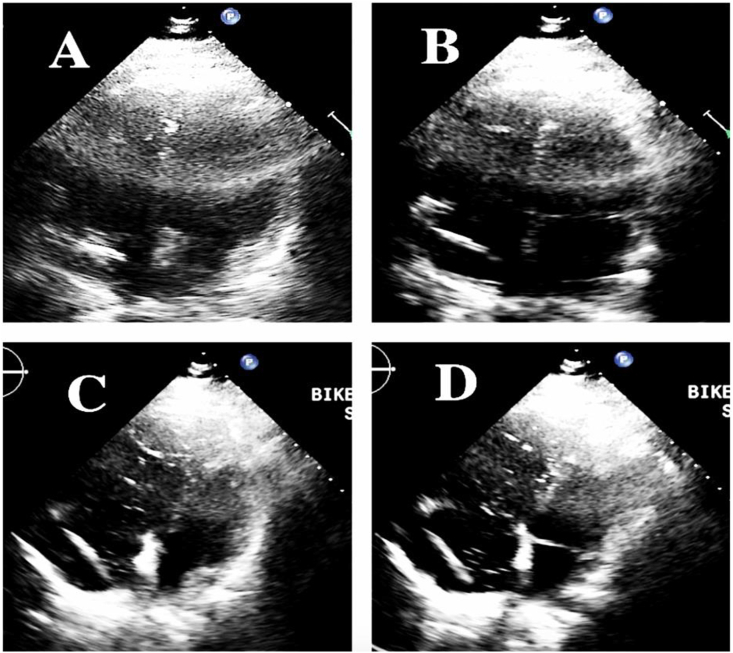
Leftward ventricular septal shift during exercise. Panels A and B show baseline echocardiographic four chamber views in diastole and systole, respectively. Panels C and D are the same views during exercise, highlighting RV dilation and a reduction in LV volume.

Pulmonary arterial hypertension is defined as mean pulmonary arterial pressure greater than 20 mmHg along with a pulmonary capillary wedge pressure less than 15 mmHg and pulmonary vascular resistance greater than 2 Wood units (WU). If left untreated, PAH leads to RV dilatation and dysfunction with eventual right heart failure. PAH-specific therapies are pulmonary vasodilators which offload right ventricular pressure by increasing forward flow to the left ventricle. Use of PAH-specific drugs may be associated with a multitude of complications, particularly in the presence of concurrent left heart disease. In these cases, pulmonary vasodilators should be used judiciously. The left ventricular end diastolic pressure (LVEDP), a measure of LV filling, is elevated in left ventricular systolic and diastolic dysfunction. Pulmonary vasodilatation leads to augmented flow to the left ventricle and further increase in LVEDP. This overload of the left ventricle can present clinically as pulmonary edema (see Fig. 1).

This can also lead to the “reverse Bernheim syndrome”. It is a phenomenon of ventricular interdependence or ventricular interaction [1]. The effect is caused by right ventricular pressure and volume overload that leads to a leftward shift of the interventricular septum. This distortion affects the left ventricular volume and function. Clinically patients show paradoxical manifestations of left sided heart failure in a backdrop of right ventricular dysfunction. This phenomenon is generally seen in patients with severe pulmonary arterial hypertension and in pulmonary embolism.

Here we report a case of a patient with pulmonary arterial hypertension who presented clinically with worsening left heart failure. After hemodynamic evaluation, his PAH-specific therapy was de-escalated to reduce the extent of pulmonary vasodilatation - the accepted standard of care based upon clinical expertise. However, his symptoms worsened upon weaning of the pulmonary vasodilators. Subsequently, it was discovered that his apparent clinical features of left ventricular failure were due to the “reverse Bernheim phenomenon”. He instead required enhancement pulmonary vasodilation. This case demonstrates a complex clinical scenario of a classical diagnostic dilemma. Uniquely in this circumstance, identical clinical manifestations can arise from two contrasting hemodynamic situations where opposing therapeutic management is needed.

## 2 Case presentation

A 70-year-old man presented with shortness of breath and recurrent syncopal episodes after walking short distances. His past medical history included pulmonary arterial hypertension, smoking related mild chronic obstructive pulmonary disease (COPD), coronary artery disease, sick sinus syndrome for which he had a permanent pacemaker placed, hypertension, diastolic heart failure and chronic respiratory failure on supplemental oxygen.

He had been diagnosed with pulmonary arterial hypertension two years prior to presentation. His initial right heart catheterization showed pulmonary artery pressure of 80/28 (48) mmHg with a pulmonary capillary wedge pressure of 14 mmHg. The pulmonary vascular resistance was calculated at 4.65 WU. For this he was started sequentially on pulmonary vasodilator therapy with oral sildenafil, ambrisentan then followed by selexipag. Six months after initiating selexipag, he developed worsening dyspnea and increasing oxygen requirement for which he was admitted to our institution.

On physical examination he was afebrile, normotensive with a blood pressure of 128/55 mmHg, heart rate of 81 beats per minute, and respiratory rate of 20 breaths per minute. Cardiac exam revealed no murmurs but present jugular venous distension. Chest auscultation was notable for bibasilar crackles. His extremities were warm and dry without evidence of edema. Orthostatic vital signs were unremarkable.

Laboratory data included hemoglobin of 10.6 mg/dL (13.0–17.0); hematocrit 32.7 mg/dL (39.0–49.0); BUN 20 mg/dL (8–23), creatinine 1.4 mg/dL (0.8–1.3), proBNP 1418 pg/mL (0–125), troponin 0.010 ng/L (0–0.04). Electrocardiogram showed normal sinus rhythm without ST or T wave abnormalities. Chest x-ray revealed a normal cardio-mediastinal silhouette with evidence of an old left basal scar with no discernible pulmonary edema.

Early in the hospital course he suffered a witnessed syncopal event. While walking in the corridor, he collapsed and became cyanotic. His heart rate at the time was noted to be 110 beats per minute and oxygen saturation 64 %. The pulse was faintly palpable. He regained consciousness after a few seconds without any altered mentation or postictal state.

## 3 Investigations

A loop recorder for cardiac rhythm assessment demonstrated short episodes of rare idioventricular rhythm not associated with the syncopal event.

Right heart catheterization was performed. This showed pulmonary artery pressures 61/24 (39) mmHg with a pulmonary capillary wedge pressure of 18 mmHg. Pulmonary vascular resistance was calculated to be 2.39 WU. Due to the presence of concurrent left heart disease and values obtained from the right heart catheterization his clinical worsening was attributed to increasing pulmonary congestion thought to be due to volume overload. Selexipag was subsequently discontinued in efforts to reduce left ventricular preload from the pulmonary vasodilatation that would result and concerns about further increasing the LVEDP and overloading the left ventricle. However, the patient clinically deteriorated after cessation of Selxipag raising concerns about an alternative etiology outside of left heart disease contributing to patient's pulmonary hypertension.

To evaluate exertional symptoms a static bicycle exercise echocardiogram was performed. During the evaluation, he became lightheaded with a sharp decline in oxygen saturation. His blood pressure significantly dropped from resting 106/60 mmHg to uninterpretable though he never lost a pulse. Review of his echocardiogram during the event revealed a dramatic right ventricular dilatation and drop in function with associated left ventricular outflow tract obstruction leading to a transient low output state. The tricuspid annular plane systolic excursion (TAPSE) had decreased from 21 to 8 mm (>17). His right ventricular dilatation and dysfunction promptly returned to baseline at rest.

## 4 Treatment

Given the above hemodynamic finding his exertional syncopal events were suggestive of Reverse Bernheim syndrome. His PAH-specific therapy was escalated by starting intravenous treprostinil therapy.

## 5 Outcome and follow-up

Distinct clinical improvement was noted after initiation and uptitration of Treprostinil. His exercise tolerance improved and syncopal episodes resolved. He returned to his baseline of WHO functional class II. A stress echocardiogram eight months later showed mild RV dilatation with no overt LVOT compression on exercise. He continues to be followed at 18 months after his initial episodes of syncope. He remains on ambrisentan, sildenafil and intravenous treprostinil for his pulmonary arterial hypertension, having achieved clinical stability.

## 6 Discussion

Bernheim syndrome was first described in 1910 by the French physiologist P.I. Bernheim [2]. The syndrome occurs due to severe left ventricular dilatation or hypertrophy leading to shifting of the interventricular septum to the right, compressing the right ventricle. His observations led to the conclusion that the signs and symptoms of right heart failure due to left ventricular hypertrophy preceded the clinical manifestation of left heart failure in left heart disease [1]. Clinically patients with Bernheim syndrome experience features of right heart failure, including distended neck veins, hepatomegaly and peripheral edema without any features of left heart failure, such as pulmonary congestion. In these cases, Bernheim argued that “right ventricular stenosis” rather than the typical dilation of the right ventricle should be considered as a cause of these symptoms. Autopsy findings of patients with these patients confirmed a physical obstruction of the right ventricular outflow tract from left ventricular dilatation or hypertrophy. This is also seen in concentric left ventricular hypertrophy associated with aortic stenosis and hypertension. Although some authors dispute its existence [3], findings consistent with Bernheim syndrome have been observed in both ante-mortem and postmortem evaluations of many patients [4,5].

Our patient suffered the exact opposite hemodynamic consequences of what Bernheim had described. This “reverse Bernheim syndrome” is also known as ventricular interdependence or ventricular interaction [6]. The hemodynamic phenomenon is caused by a leftward movement of interventricular septum due to severe right ventricular dilatation, resulting in compression of the left ventricular cavity [7]. The resultant LVOT obstruction leads to systolic dysfunction with reduced ejection from the left ventricle, as well as diastolic dysfunction due to increases left ventricular filling pressure seen as an increased LVEDP. This phenomenon of ventricular interdependence describes that right heart dysfunction can clinically manifest as new onset or worsening left heart failure. It can be observed in both a normal as well as a diseased left ventricle. In the reverse Bernheim syndrome, the left ventricular diastolic function is more likely to be affected than the systolic function [8].

Several authors have reported presence of left sided diastolic dysfunction in advanced PAH patients with or without associated left heart systolic disease [[9], [10], [11], [12]]. Tonelli et al. noted that left ventricular diastolic dysfunction is common and correlates with the severity of PAH [11]. Other authors noted similar outcomes including that left ventricular diastolic dysfunction in PAH patients predict functional capacity and clinical worsening [13]. Galie et al. found improved echocardiographic markers of diastolic dysfunction (E/A ratio and peak E velocity) after therapy in PAH patients [14].

Impaired left ventricular diastolic relaxation has been attributed to a multitude of causes, predominantly due to leftward displacement of the interventricular septum. Louie and Brundage demonstrated the effect of distortion of left ventricular geometry resulting from displacement of the interventricular septum towards the left ventricular cavity in PAH [10]. Functionally this leads to prolonged isovolumetric relaxation and significant reduction in early diastolic cavity expansion. This leftward shift was more pronounced in end systole and early diastole [10].

The reverse Bernheim phenomenon in advanced PAH, as seen in our patient, is an extreme spectrum of this condition. Even in a chronically dilated right ventricle, the restraining influence of the pericardium may result in abrupt leftward shift of interventricular septum due to an acute rise in pulmonary artery pressures during exercise. This will lead to severe distortion of left ventricular geometry, affecting its systolic and diastolic function. The consequent clinical effects include syncope from reduced left ventricular ejection and pulmonary edema from impaired left ventricular filling.

Thus far very few cases of the Reverse Bernheim Phenomenon have been described in literature. Marcus et al. used nuclear MRI to analyze left ventricular and right ventricular volumes and filling pattern in 12 severe PAH patients. The study demonstrated that the bowing of the interventricular septum into the left ventricle during diastole was associated with slower filling of the left ventricle compared to the right ventricle. This resulted in marked reduction in left ventricular stroke volume by the Frank-sterling mechanism [12]. Schena and coworkers demonstrated that chronic right ventricular overload induces left ventricular filling impairment due to septal leftward shift in patients with advanced COPD and cor pulmonale [15]. Similar ventricular interdependence was also illustrated by Vizza and colleagues in end stage pulmonary diseases where the occurrence of left ventricular dysfunction was correlated to presence of right ventricular dysfunction [16].

In our patient, the stress echocardiogram was able to capture a dramatic RV dilatation and septal bulge leading to a transient sharp drop in left ventricular ejection, causing symptoms of lightheadedness and pre-syncope. After initiation of Treprostinil and subsequent improvement in his pulmonary artery pressures and right ventricular function, the patient showed resolution of his syncopal episodes. A repeat stress echocardiogram after treatment was able to demonstrate this improvement in exercise-induced right ventricular dilatation and septal shift.

The uniqueness of our case lies in the fact that the patient's echocardiogram did not reveal any features of reverse Bernheim effect at rest. To our knowledge, D'Avila et al. have reported the only other case of exercise-induced reverse Bernheim phenomenon prior to this publication [17]. Similar to our patient, D'Avila et al. confirmed the sudden rise in pulmonary artery pressures and right ventricular dilation with subsequent leftward shift of interventricular septum using a stress-echocardiogram treadmill test [17]. The exercise-induced syncope in severe PAH occurred due to the reverse Bernheim phenomenon in both cases.

The more frequent scenario of developing or worsening diastolic heart failure in PAH is considered secondary to increased flow into the left heart. The rise in left sided filling pressures can be ameliorated by reducing pulmonary flow. Common wisdom in this scenario would be to reduce the amount of pulmonary vasodilation, however, the elevated left ventricular filling pressure due to Bernheim effect can only be alleviated by reducing the size of the right ventricle which paradoxically requires augmentation of pulmonary vasodilators. Moreover, the severe form of the reverse Bernheim effect will affect left ventricular systolic function as well. The de-escalation of pulmonary vasodilator in our patient exacerbated this hemodynamic phenomenon, worsening his symptoms and syncopal events. This distinction is critical because conflicting hemodynamic situations yielding identical clinical manifestations where the required interventions are diametrically opposite.

In conclusion, the reverse Bernheim phenomenon is an infrequent yet noteworthy manifestation of pulmonary arterial hypertension. As PAH is becoming an increasingly recognized entity, with early diagnosis and treatment, both specialists and community physicians will likely come across PAH-related issues in their patients. The important clinical lesson of this problem is paramount for safe and appropriate patient care. If not detected early and intervened appropriately, the consequences may be fatal.

## CRediT authorship contribution statement

**Evan Wasserman:** Writing – review & editing. **Luis Wulff:** Writing – original draft. **Debabrata Bandyopadhyay:** Writing – original draft, Supervision. **Ricardo Restrepo-Jaramillo:** Writing – original draft, Supervision. **Raheel Qureshi:** Writing – original draft, Supervision. **Jose Herazo-Maya:** Writing – original draft, Supervision. **Monirul Islam:** Writing – original draft, Supervision.

## Learning Points

Reverse Bernheim phenomenon is an uncommon manifestation of advanced pulmonary arterial hypertension which occurs due to severe dilatation of the right ventricle, resulting in leftward bulging of interventricular septum and subsequent distortion of left ventricular geometry. The consequent effect includes compromised left ventricular diastolic and systolic function.

· This anomalous hemodynamic phenomenon results in clinical manifestations of left heart failure in setting RV dysfunction.
· Paradoxically, escalation of PAH-specific therapy is indicated in this situation.
· A high index of suspicion is required for early detection of this devastating complication and prompt appropriate intervention.

## Declaration of competing interest

The authors declare that they have no known competing financial interests or personal relationships that could have appeared to influence the work reported in this paper.
